# T76. INVESTIGATION OF NAV1.1 POSITIVE MODULATOR EFFECTS ON FAST SPIKING INTERNEURONS IN SOMATOSENSORY CORTEX SLICES

**DOI:** 10.1093/schbul/sby016.352

**Published:** 2018-04-01

**Authors:** Piszar Ildiko, Just Stefan, Draheim Henning

**Affiliations:** Boehringer Ingelheim

## Abstract

**Background:**

GABAergic inhibition is essential for normal cortical function as it serves the purpose of proper excitation/inhibition (E/I) balance in many circuits. In contrast, inappropriate interneuron signaling leads to E/I imbalance, reduced gamma oscillations in EEG measurements and has serious behavioural consequences. Among the heterogeneous group of GABAergic cells, fast-spiking, parvalbumin positive interneurons (FS-PV+) play a key role in the generation and maintenance of gamma oscillations. E/I imbalance due to interneuron dysfunction has been implicated in the pathophysiology of various psychiatric disorders.

Cognitive impairment has been associated with altered gamma oscillation in schizophrenia and there is accumulating evidence for involvement of FS-PV+ interneuron deficit in the disease. Hypofunction of FS-PV+ neurons leads to disinhibition of pyramidal cells which cause network desynchronization. Therefore, it is hypothesized that activation of these neurons could restore high-frequency oscillations and consequently improve cognitive functions. However selective modulation of different interneuron types is still challenging due to limited number of known cell type specific targets.

**Methods:**

A possible starting point for the treatment could be pharmacological activation of voltage gated sodium channels (Nav) which have a pivotal role in action potential initiation. Of the four subtypes of Nav channels expressed in the CNS Nav 1.1 comprises the majority of the sodium current in FS-PV+ but not in pyramidal neurons. Based on this we looked for selective Nav 1.1 activators and found a recently published promising compound (Compound 3a, see Crestey et al, 2015) which has been shown to increase the electrical activity of FS-PV+ interneurons in the CA1 area of the hippocampus. However, the dysfunction in information processing found in schizophrenic patients is not only restricted to the hippocampus and high-order association cortices but also influences the sensory cortex. Thus, our aim was to explore the effect of the selective Nav 1.1 positive modulator Compound 3a on FS interneurons in the mouse somatosensory cortex. We performed whole-cell patch clamp recordings from mouse cortical brain slices and recorded the electrical activity of single FS cells before and after the drug application.

**Results:**

Surprisingly the excitatory effect of the compound 3a could only partly be confirmed in the way that positive modulation of Nav1.1 in terms of action potential number and threshold only takes place under particular conditions, i.e. at physiological temperature and under specific ion compositions of the recording solutions

**Discussion:**

The discrepancy of our results from published data might be attributed to the different experimental conditions such as recording temperature and ionic composition of solutions and highlight the importance of selecting near physiological conditions during brain slice patch clamp experiments.

